# Post-interview Thank-you Communications Influence Both Applicant and Residency Program Rank Lists in Emergency Medicine

**DOI:** 10.5811/westjem.2019.10.44031

**Published:** 2019-12-09

**Authors:** Corlin Jewell, Tillman David, Aaron Kraut, Jamie Hess, Mary Westergaard, Benjamin H. Schnapp

**Affiliations:** University of Wisconsin School of Medicine and Public Health, Berbee Walsh Department of Emergency Medicine, Madison, Wisconsin

## Abstract

**Introduction:**

The National Residency Matching Program (NRMP) allows post-interview contact between residency applicants and residency programs. Thank-you communications represent one of the most common forms, but data on their value to applicants and program directors (PD) are limited. The objective of this study was to assess the effect of thank-you communications on applicant- and residency-program rank lists.

**Methods:**

Two anonymous, voluntary surveys were sent after the 2018 NRMP Match, one to applicants who were offered an interview at a single academic site in the 2017–2018 Match cycle, and one to EM PDs nationwide. The surveys were designed in conjunction with a nationally-recognized survey center and piloted and revised based on feedback from residents and faculty.

**Results:**

Of 196 residency applicants, 97 (49.5%) responded to the survey. Of these, 73/95 (76.8%) reported sending thank-you communications. Twenty-two of 73 (30%) stated that they sent thank-you communications to improve their spot on a program’s rank list; and 16 of 73 (21.9%) reported that they changed their rank list based upon the responses they received to their thank-you communications. Of 163 PDs, 99 (60.7%) responded to the survey. Of those PDs surveyed, 22.6% reported that an applicant could be moved up their program’s rank list and 10.8% reported that an applicant could move down a program’s rank list based on their thank-you communications (or lack thereof).

**Conclusion:**

The majority of applicants to EM are sending thank-you communications. A significant minority of applicants and PDs changed their rank list due to post-interview thank-you communications.

## INTRODUCTION

Applying for residency in emergency medicine (EM) is a highly consequential process that fourth- year medical students undergo every year in order to determine where they will undertake specialty training.^1^ This, in many cases, sets the direction for the rest of their career. The current application process demands significant time and energy and, for the average applicant, costs more than $8000.^2^ On average, each EM residency applicant sends out applications to 41 programs and attends 13 interviews.^3^ After each interview day, many applicants set aside time for yet another task: sending thank-you communications to those programs at which they interviewed.^4^

The National Residency Matching Program (NRMP) allows post-interview contact between applicants and programs but requires that both parties follow a specific code of conduct.^5^ Recognizing that applicants represent a potentially vulnerable population, the code states that programs may not engage in communication that reveals or influences rank lists. Despite this, post-interview communication has been shown on multiple occasions to influence how an applicant ranks programs.^4^,^6^,^7^ Previous work has shown that most applicants were contacted in some form by programs and that they were glad to receive such communication.^8^ Despite the absence of any clear evidence in favor of the practice, medical students applying to EM residency are usually advised by clerkship directors and faculty mentors to communicate their thanks to programs.^9^ However, it is currently unknown whether the practice benefits applicants or programs.

While previous studies have examined the impact of post-interview contact (including return visits to an institution, or “second looks,” phone/email correspondence, etc) from residency programs, no previous work has focused solely on the impact of post-interview thank-you communications in EM on both applicant and program rank lists. The goal of this study was to define current applicant thank-you communication practices, how these thank-you communications are perceived by program directors (PD), and whether applicants are influenced by responses to them.

## METHODS

### Study Design

This was a cross-sectional study that surveyed two separate but complementary populations. One survey was administered to all applicants to our institution’s EM residency program who were offered an interview in the 2017–2018 NRMP cycle. A second survey was administered to a list of EM PDs compiled from information abstracted from a database created by members of the Council of Emergency Medicine Residency Directors (CORD). This data was further improved upon by assessing publicly available PD contact information obtained from EM residency program websites. We used survey methodology because the questions being studied could not be adequately answered by looking at reported Match data alone; so we sought to explore the opinions of the applicants and PDs.^10^

### Study Setting and Population

The applicant arm of the study was conducted at a three-year academic EM residency program that currently offers 12 postgraduate year (PGY)-1 positions. All applicants who applied to our program during the 2017–2018 NRMP application cycle and received an offer to interview (regardless of whether they accepted the interview offer) were eligible to participate in the applicant arm of the study. All current allopathic EM PDs nationwide were eligible for the PD arm. Of the 220 EM programs that participated in the 2017–2018 NRMP Match, 163 had contact information from their PD available from the CORD data and/or from their program’s website and were emailed the survey.^1^ The decision to deploy the survey only to allopathic PDs was done to keep the two surveyed populations complementary to each other, as the study institution did not offer an interview to any osteopathic students during this application cycle.

**Educational Research Capsule Summary**

What do we already know about this issue?

*The National Residency Matching Program allows post-interview contact between residency applicants and programs and thank-you communications represent one of the most common forms*.

What was the research question?

What is the effect of thank-you communications on applicant and residency-program rank lists?

What was the major finding of the study?

*Many applicants and program directors changed their rank list based on post-interview thank-you communications and the responses to them*.

How does this improve population health?

*This study helped to provide information on the utility on sending thank-you communications, a common convention in the post-interview process*.

### Survey Development

To our knowledge, there are no prior surveys with validity evidence that answered the questions we sought to explore in our study. Therefore, two new instruments were created. The survey instruments used in our study were designed in conjunction with our university’s survey center. This research organization operates with a budget of over $6 million annually and has a well-established history of surveying physicians by web.^11^ The survey instrument for the applicant arm (Appendix 1) was designed to incorporate multiple-choice and Likert-scale questions to assess whether applicants sent thank-you communications, what form these thank-you communications took (email, paper, etc.), why they sent them, and what influence the responses to them had in the post-interview period. The survey instrument of the PD arm (Appendix 2) also incorporated multiple-choice and Likert-scale questions to assess how thank-you communications from applicants were received and how they affected their program’s rank list (if at all). The survey instruments were first developed and then reviewed and edited by EM education research faculty at our institution. This process of review was undertaken to enhance the content validity of the surveys. We performed two separate pilot surveys to attempt to increase clarity and reduce response biases. The applicant instrument was piloted on current PGY-1s at our training program, as they were closest to the survey’s target population. The PD instrument was piloted on EM education faculty. We made final revisions based on comments from the pilot populations prior to survey distribution to enhance response process validity.

### Study Protocol

Participants in both arms were invited to complete an anonymous and voluntary survey via the provided email addresses in their Electronic Residency Application Service (ERAS) application (applicants) and a database our program keeps of current residency PDs. In an effort to reduce the potential for influencing the responses from applicants or changing their own NRMP rank list, the applicant arm of the survey was distributed after the 2018 NRMP Match date. The survey was administered online using Qualtrics (Provo, UT). The overall response rate of both surveys used the second definition of response rate provided by the American Association for Public Opinion Research.^12^ Participants were allowed to skip any question they did not wish to answer. Two reminder emails were sent at approximately two-week intervals. The study design was determined to be exempt by our institutional review board.

We calculated descriptive statistics using Qualtrics. A wave analysis following the final reminder email to assess for nonresponse bias was calculated on the applicant survey for the questions, “Did you send thank-you communications to any programs after interview day?,” and “Did you ever adjust your rank list based on how programs responded to these thank-you communications?” A second-wave analysis following the final reminder email was performed on the PD survey for the questions of “Does an applicant ever move UP your rank list because of their thank-you communication following the interview day?” and “Does an applicant ever move DOWN your rank list because of their thank-you communication or lack of thank-you communication following the interview day?” These questions were selected as they were felt to have the most impact for readers. These wave analyses assumed a response of YES=1 and NO=0 for each question. For both sets of analyses, the responses following the final reminder email were used as a proxy for nonresponders.^13^

## RESULTS

### Applicant Survey

**Figure 1:**
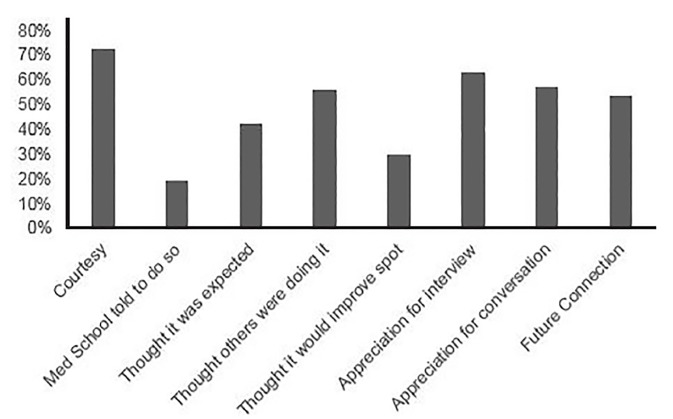
Reasons reported by applicants for sending thank-you communications.

Overall, 97/196 (49.5%) applicants responded to the survey. Given that not all applicants responded to each question, the percentages reported are based on the total number of responses for each question individually. Of the 97 applicants who responded, the majority (76.8%) reported sending thank-you communications to at least one program. The nonresponse bias (NRB) for this question was calculated via wave analysis at 0.088. Nearly all of them communicated their thanks via email (87.7%), while the remaining applicants sent written letters. None of the responders communicated their thanks via phone call. Figure 1 shows the reasons applicants gave for communicating their thanks (respondents could supply more than one answer). A total of 19.2% of applicants responded that they received responses to their thank-you communications “almost always,” and a further 56.2% received responses either “often” or “sometimes.” The majority of applicants (56.2%) reported spending at least 15 minutes on their thank-you communications per program, and 8.3% reported spending greater than 45 minutes per program. Finally, more than a fifth (21.9%) reported that they changed their rank list based on the responses they received to their thank-you communication (NRB = 0.043).

### Program Director Survey

**Figure 2:**
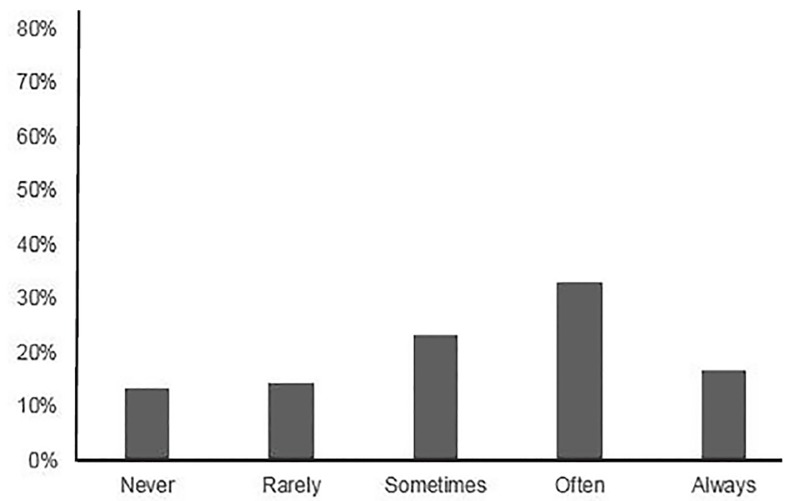
Reported rate of tailored responses to thank-you communications by program directors.

Of the PDs surveyed, 99/163 (60.7%) responded at least partially. As with the applicant survey, the percentages are reported based on the total number of responses for each question. Of these, 39.5% reported responding to thank-you communications from applicants “often” or “always.” Nearly half of them (45/91; 49.5%) also reported personalizing their responses to individual applicants (Figure 2). When asked if applicants moved up their program’s rank list based on thank-you communications, 21/93 (22.6%) responded “yes” (NRB = 0.018). Of the PDs who answered “yes,” 14/21 (66.7%) reported that an applicant could expect to move up six or more positions on the rank list.

**Figure 3:**
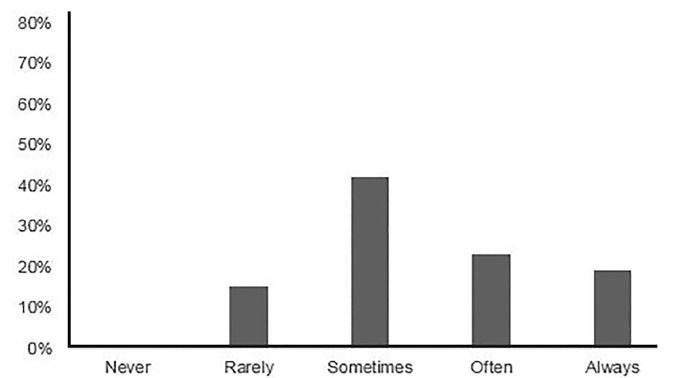
Reported importance placed by program directors on the specific content of thank-you communications from applicants.

A total of 10/93 (10.8%) reported that an applicant could potentially move down on their rank list based on their thank-you communications (or lack thereof) (NRB = 0.048). Of these 10 PDs, 6/10 reported that applicants could move down six or more positions. More than half of the PDs (22/26; 84.6%) who stated that applicants could change position on the rank list based on their thank-you communication considered specific content of the thank-you communication to be important (Figure 3). In this same cohort of PDs, most (18/26; 69.2%) reported that the form of the thank-you communication (email, written, etc.) “rarely” or “never” affected how an applicant moved positions on their program’s rank list. However, the majority of PDs (61/78; 78.2%) reported that they preferred email, while the remainder preferred written communication (21.8%).

## DISCUSSION

Within the respondents to our survey (49.5% of the survey population), who represent a single three-year academic residency program in the Midwest, most applicants reported sending thank-you communications. Within this same group, those who sent thank-you notes reported changing their rank lists based on the program’s response to their notes. The most common reason for sending these thank-you communications was reported to be courtesy. Although this rationale may demonstrate positive personal character and sending thank-you communications may even portend a favorable residency outcome,^13^ a large number of applicants believed that sending thank-you communications would have a positive impact on their relationship and standing with a residency program. While it is known that the majority of residency applicants are sending thank-you communications, a reported effect on both applicant and program rank lists in EM has not been shown previously.

The strategy of writing thank-you communications to programs to boost an applicant’s competitiveness seems at least somewhat effective given that the specific content of thank-you communications is highly rated as a factor affecting rank list movement by PDs. There are no official “best practices” regarding how post-interview thank-you communication should be formatted apart from the mandate from the NRMP that “Program Directors shall not solicit or require post-interview communication from applicants, nor shall program directors engage in post-interview communication that is disingenuous for the purpose of influencing applicants’ ranking preferences.”^5^ Applicants have few resources to assist them, with unofficial “Application Guides”^14^–^16^ from faculty and blogs, which rely on anecdotal evidence, serving as the primary guideposts. It is interesting to note that, despite the Match Code of Conduct policy of not soliciting or requiring post-interview communication form applicants, it appears that not writing thank-you communications is used in the rank list decision-making by programs, which could be construed as a policy violation.

The amount of applications and interviews per EM residency applicant is increasing, with the average allopathic United States senior applying to 41 programs and attending 13 interviews.^2^ Due to the low likelihood that any individual thank-you communication influences a desired program’s rank list enough to turn an applicant’s non-matchable rank into a matchable one, the potential time-cost of thank-you communications initially appears unfavorable for the applicant compared to the small likelihood of potential benefits. However, a significant minority of PDs reported that an applicant’s thank-you communication could significantly affect the applicant’s rank position. Therefore, our data suggest that an applicant’s reported goal of writing thank-you communications to give their application a boost on the rank list is grounded in some truth. Given the high stakes of the application process and the substantial time, financial, and personal investment involved in the residency application process, candidates will likely continue to send thank-you communications if there is any possibility of influencing their rank list position. Programs that do not consider thank-you communications when adjusting their rank list may potentially save applicants time and effort if they are forthcoming about discouraging thank-you communications in the post-interview period.

Nearly a quarter of applicants reported changing their rank lists due to the responses they received from thank-you communications. This proportion is similar to other previously published work on post-match applicant surveys.^7^,^17^,^18^ Our survey did not specifically investigate the content of these responses and how residency applicants were using them to make rank list decisions nor how the absence of responses affected an applicant’s decision to rank a program. It is possible that receiving these responses reinforces connections made during the brief interview day and could subconsciously make applicants feel as if they “fit in” better with a particular program.

“Fit” has been demonstrated previously as one of the most important factors in program selection by applicants.^19^ It is also possible that applicants perceive responses to thank-you communications as an indicator that they were seen favorably by the program, despite the fact that many PDs are responding to the majority of the thank-you communications sent to them. Although our study did not specifically address whether a PD was more likely to send a response to thank-you communications to a competitive applicant over a non-competitive one, this question could represent an avenue of further investigation. Conversely, if applicants are changing their rank lists based on how thank-you communications affect their perceived likelihood to match at a given program, this suggests a potentially concerning misunderstanding of the stable marriage algorithm used for the NRMP Match, another potential avenue for further research.

There is also a population of EM PDs identified by this study who view applicants that do not send thank-you communications unfavorably. It is possible that programs take a lack of thank-you communications as a statement of disinterest from the applicant, which is supported by our data from the PD survey. Given the variation in how thank-you communications are received by programs, establishing more consistent standards around disclosure of how post-interview contact may or may not affect their chances of matching may benefit applicants to EM.

## LIMITATIONS

The applicant arm of the study represented responders to a single three-year academic center in the Midwest. Therefore, certain applicants, such as those desiring community/county programs or a different geographic region, may not be represented in our data, which could introduce bias. As discussed above, not all allopathic EM PDs were represented in the PD arm of the study, as a number of current PDs’ contact email addresses were not available or current; therefore, they were not included in the study. Use of the survey format, although overall appropriate for the questions being studied, did not allow for two-way communication and clarification of responses. Because we did not collect demographics in our survey to preserve anonymity, we could not determine whether specific factors, such as age or geography, influenced applicants’ propensity to write thank-you communications or PD responses to thank-you communications. However, not collecting demographic data had the side effect of making it more difficult to assess for nonresponse bias.

Although we collected data from a reasonably large number of applicants, the study period was only one year and from a single institution; thus, it is possible that respondents differed systematically from non-respondents, which, by definition, increases the potential for nonresponse bias. Questions, such as those specifically asking how many spots up or down an applicant moves on their program’s rank list may be limited by availability and recall bias. The response rate, particularly that of the applicant arm, was low, subjecting the study to potential bias. However, this is a well-recognized problem with online surveys targeting physician populations and our response rates are in line with many similar online surveys based on previously published data.^11^,^20^,^21^ The wave analysis did not show a considerable amount of calculated NRB in the selected questions, which suggests that nonresponders did not differ significantly from responders to the surveys. However, it is possible that there is some degree of nonresponse bias present as proxies (third-wave responders) were used for nonrespondents.^13^

## CONCLUSION

The majority of applicants to emergency medicine are sending thank-you communications to programs, although a considerable portion (>20%) do not send any. Based on our data a small but notable portion of both applicants and programs are willing to change their rank lists based on thank-you communications and the responses to them. Clear “best practices” are not defined by this study; however, it seems that emailed thank-you communications with attention to well-crafted content are seen favorably by a subset of PDs. Future work could focus on establishing best practices for applicants and programs and further elucidating the causes of practice variability in thank-you communications.

## Supplementary Information
