# Supplementary material for: Post-interview Thank-you Communications Influence Both Applicant and Residency Program Rank Lists in Emergency Medicine
Source: West J Emerg Med. 2019 Dec 9;21(1):96–101. doi: 10.5811/westjem.2019.10.44031 (PMC6948692; doi:10.5811/westjem.2019.10.44031)
Supplement: Supplementary file 1 [file wjem-21-96-s001.pdf]

Default Question Block

We are interested in how and why residency applicants are communicating their ***thanks to programs after interview day*** and how they are perceived and used by programs when creating their rank list.

Please note: these questions refer specifically to ***post-interview thank-you communications*** and the responses to these thank-you communications.

While answering these questions, you **should not consider subsequent correspondence** between yourself and the program (such as communicating where a program resides on your rank list). This survey should take 1-2 minutes to complete.

Block 1

Did you send thank-you communications to any programs after interview day?

- ○ Yes
- ○ No

What format did you use to say thank-you?

- ○ E-Mail
- ○ Phone Call
- ○ Written Letter

Why did you decide to communicate your thanks following interview day? Please select all that apply.

- ☐ Courtesy
- ☐ Medical school told me to
- ☐ Thought program would expect it
- ☐ Thought other applicants were doing it
- ☐ Thought it would improve my spot on the program's rank list

- [ ] Wanted to express appreciation for being offered interview
- [ ] Wanted to express appreciation for the conversation that occurred
- [ ] Wanted to cultivate a future connection
- [ ] Other

Why did you decide to communicate your thanks following interview day?

Which individuals did you consistently use thank-you communications for? Please select all that apply.

- [ ] Assistant Program Directors
- [ ] Department Chairs
- [ ] Faculty Interviewers
- [ ] Program Directors
- [ ] Residency Coordinators
- [ ] Residents (e.g. hosts, interviewers, etc.)
- [ ] None

How many thank-you communications would you send, on average, to a single program?

- ○ 0-1
- ○ 2-3
- ○ 3-4
- ○ >4

How much time did you spend, on average, on thank-you communications to each program?

- ○ <15 minutes
- ○ 15-30 minutes
- ○ 30-45 minutes
- ○ >45 minutes

How often did you receive responses to your thank-you communications?

- Almost always
- Often
- Sometimes
- Seldom
- Never

Did you ever adjust your rank list based on how programs responded to these thank-you communications?

- Yes
- No

Did the time between interviews and submitting your rank list increase or decrease your anxiety about the match process?

- Increase
- Decrease
- Neither

Block 2

Powered by Qualtrics
