# Supplementary material for: Post-interview Thank-you Communications Influence Both Applicant and Residency Program Rank Lists in Emergency Medicine
Source: West J Emerg Med. 2019 Dec 9;21(1):96–101. doi: 10.5811/westjem.2019.10.44031 (PMC6948692; doi:10.5811/westjem.2019.10.44031)
Supplement: Supplementary file 2 [file wjem-21-96-s002.pdf]

Default Question Block

This survey is part of a study designed to look at how and why residency applicants are communicating their thanks to programs following their interview day as well as investigating how program directors, such as yourself, perceive and use these thank-you communications. Please note: these questions refer specifically to ***post-interview thank-you communications only*** and ***should not include subsequent correspondence between yourself and the applicant***. This survey should take 1-2 minutes to complete.

Block 1

About how many applicants would you estimate sent thank-you communications after their interview?

- 0-20%
- 20-40%
- 40-60%
- 60-80%
- 80-100%

Does an applicant ever move UP your rank list because of their thank-you communication following the interview day?

- Yes
- No

How much does an applicant generally move UP your rank list because of their thank you communication?

- 1-5 spots
- 6-10 spots
- 11-15 spots

- 16-20 spots
- 20+ spots

Does an applicant ever move DOWN your rank list because of their thank-you communication or lack of thank-you communication following the interview day?

- Yes
- No

How much does an applicant generally move DOWN your rank list because of their thank you communication or lack of thank-you communication?

- 1-5 spots
- 6-10 spots
- 11-15 spots
- 16-20 spots
- 20+ spots

Does the specific content of an applicant's thank-you communication matter for moving an applicant on your rank list?

- Never
- Rarely
- Sometimes
- Often
- Always

Does the specific format (e.g. handwritten, email, phone call) of an applicant's thank-you communication matter for moving an applicant on your rank list?

- Never
- Rarely
- Sometimes
- Often
- Always

By what method do you prefer applicants communicate their thanks?

- E-mail
- Written Letter
- Phone call

How often did you respond to thank-you communications from applicants?

- Never
- Rarely
- Sometimes
- Often
- Always

How often did you personally tailor your responses to the content in these thank-you communications?

- Never
- Rarely
- Sometimes
- Often
- Always

Powered by Qualtrics
